# Ethnic differences in the age-related distribution of serum prostate-specific antigen values: A study in a Taiwanese male population

**DOI:** 10.1371/journal.pone.0283040

**Published:** 2023-03-16

**Authors:** Tsung-Hsun Tsai, Ta-Wei Chu, Tien-Huang Lin, Teng-Fu Hsieh, Chi-Cheng Chen, Hsin-Ho Liu, Yuan-Chieh Chuang, Chia-Wen Lin, Shang-Sen Lee

**Affiliations:** 1 Department of Urology, Taichung Tzu Chi Hospital, Buddhist Tzu Chi Medical Foundation, Taichung, Taiwan; 2 School of Medicine, Tzu Chi University, Hualian, Taiwan; 3 Department of Obstetrics and Gynecology, Tri-Service General Hospital, National Defense Medical Center, Taipei, Taiwan; 4 MJ Health Screening Center, Taipei, Taiwan; 5 MJ Health Research Foundation, Taipei, Taiwan; 6 Department of Urology, Tri-Service General Hospital, National Defense Medical Center, Taipei, Taiwan; Kyung Hee University School of Medicine, REPUBLIC OF KOREA

## Abstract

This study investigates age-specific prostate-specific antigen (PSA) distributions in Taiwanese men and recommends reference ranges for this population after comparison with other studies. From January 1999 to December 2016, a total of 213,986 Taiwanese men aged above 19 years old without history of prostate cancer, urinary tract infection, or prostate infection were recruited from the Taiwan MJ cohort, an ongoing prospective cohort of health examinations conducted by the MJ Health Screening Center in Taiwan. Participants were divided into seven age groups. Simple descriptive statistical analyses were carried out and quartiles and 95th percentiles were calculated for each group as reference ranges for serum PSA in screening for prostate cancer in Taiwanese men. Serum PSA concentration correlated with age (r = 0.274, p<0.001). The median serum PSA concentration (5th to 95th percentile) ranged from 0.7 ng/ml (0.3 to 1.8) for men 20–29 years old (n = 6,382) to 1.6 ng/ml (0.4 to 8.4) for men over 79 years old (n = 504). The age-specific PSA reference ranges are as follows: 20–29 years, 1.80 ng/ml; 30–39 years, 1.80 ng/ml; 40–49 years, 2.0 ng/ml; 50–59 years, 3.20 ng/ml; 60–69 years, 5.60 ng/ml; 70–79 years, 7.40 ng/ml; over 80 years, 8.40 ng/ml. Almost no change occurred in the median serum PSA value in men 50 years old or younger, while a gradual increase was observed in men over 50. Taiwanese men aged 60 years above showed higher 95th percentile serum PSA values compared to Caucasian men and men in other Asian countries but were closer to those of Asian American and African American men. Results indicate significantly different PSA levels correlating to different ethnicities, suggesting that Oesterling’s age-specific PSA reference ranges might not be appropriate for Taiwanese men. Our results should be further studied to validate the age-specific PSA reference ranges for Taiwanese men presented in this study.

## Introduction

Prostate cancer is one of the most common malignancies in men worldwide. Prostate-specific antigen (PSA) is presently the most widely used tumor marker for prostate cancer diagnosis, staging and treatment follow-up [1, 2]. PSA levels are expected to increase with age as a result of hypertrophied prostate. Therefore, using the current standard single cut-off of 4.0 ng/ml, might not be appropriate for all age groups. Age-adjusted PSA levels might help in increasing the sensitivity rate of prostate cancer detection in younger men and in decreasing unnecessary biopsies in older subjects [3–6].

In 1993, Oesterling et al. [7] first introduced age-specific reference ranges for serum PSA for Caucasian Americans. However, the rate of prostate growth and the volume of prostate-producing PSA is known to vary among ethnic groups [8, 9], and Oesterling’s age-specific PSA reference ranges may not be generally applicable to all populations.

Many studies about age-adjusted PSA values have been conducted for African Americans [10], Koreans [11], Japanese [8], and Chinese [12–14], and even for Taiwanese [15], but no large-scale study has been conducted among Taiwanese.

This study determines age-specific PSA distributions in Taiwanese men, with the goal of offering optimal values for detecting the upper limit of serum PSA levels based on a large study cohort.

## Methods

This study recruited 213,986 Taiwanese men aged above 19 years old without history of prostate cancer, urinary tract infection, or prostate infection who had undergone a routine health checkup from the MJ Health Screening Center in Taiwan from January 1999 through December 2016 [16]. The MJ Health Database only includes participants who provided informed consent. The study protocol was approved by the Ethics Committee of Taichung Tzu Chi Hospital, Buddhist Tzu Chi Medical Research Foundation (REC 109–54).

Participants were divided into seven age cohorts based on age at the time of the most recent screening: 20–29 years of age (6,382 individuals), 30–39 (33,555), 40–49 (78,912), 50–59 (50,229), 60–69 (31,252), 70–79 (13,152), and above 79 (504).

The PSA values were analyzed using Stata version 14.2 (Stata, College Station, TX, USA). The association between serum PSA levels and age was measured using Pearson product-correlation coefficients [17]. Descriptive statistics including the mean, median, 5th, 25th, 75th, and 95th percentiles of the PSA level distribution were calculated for each 10 year age group cohort [18].

## Results

Among the 213,986 participants, the mean PSA level was around 1.21ng/ml. Table 1 shows the mean serum PSA concentration and median, 5th, 25th,75th and 95th percentiles for each age cohort. The median PSA value (95th percentile range) was 0.70 ng/ml (1.80) for men 20–29 years old; 0.70 ng/ml (1.80) for men 30–39 years old; 0.70 ng/ml (2.0) for men 40–49 years old; 0.80 ng/ml (3.2) for men 50–59 years old; 1.10 ng/ml (5.60) for men 60–69 years old; 1.50 ng/ml (7.40) for men 70–79 years old and 1.60 ng/ml (8.40) for men over 79 years old Table 1. The serum PSA values were correlated with age (r = 0.274, p<0.001; 95% CI 0.270 to 0.278).

**Table 1:** Serum PSA according to age in 213,986 Taiwanese men.

Age (yr)	N	Mean	SD	Serum PSA (ng/ml) Percentile Value				
				5^th^	25^th^	Median	75^th^	95^th^
20–29	6,382	0.82	0.69	0.30	0.50	0.70	1.00	1.80
30–39	33,555	0.84	0.75	0.30	0.50	0.70	1.00	1.80
40–49	78,912	0.89	0.82	0.30	0.50	0.70	1.10	2.00
50–59	50,229	1.20	1.54	0.30	0.50	0.80	1.40	3.20
60–69	31,252	1.91	4.45	0.30	0.70	1.10	2.10	5.60
70–79	13,152	2.58	5.48	0.30	0.80	1.50	2.90	7.40
80 ↑	504	2.85	4.54	0.40	0.90	1.60	3.25	8.40
Total	213,986	1.21	2.44	0.30	0.50	0.80	1.30	3.30

Abbreviation: PSA: prostate-specific antigen.

The results show only small differences in the median and 95th percentile PSA values among men younger than 50 years of age, but significant increases were found at higher ages. In those aged 20–59, 1.48% (2,510/169,078) had serum PSA levels greater than 4.0 ng/ml, as opposed to 11.38% (5,112/44,908) of men above 59 years old. Table 2 summarizes the proportions of men with various PSA values based on age.

**Table 2:** Proportion of men with various serum PSA levels according to age.

Age (yr)	N	Serum PSA Level (ng/ml)					
		<2.5		2.5–4		>4	
20–29	6,382	6,254	97.99%	86	1.35%	42	0.66%
30–39	33,555	32,840	97.87%	453	1.35%	262	0.78%
40–49	78,912	76,575	97.04%	1,611	2.04%	726	0.92%
50–59	50,229	46,007	91.59%	2,742	5.46%	1,480	2.95%
60–69	31,252	24,907	79.70%	3,425	10.96%	2,920	9.34%
70–79	13,152	9,131	69.43%	1,917	14.58%	2,104	16.00%
80 ↑	504	330	65.48%	86	17.06%	88	17.46%
Total	213,986	196,044	91.62%	10,320	4.82%	7,622	3.56%

Abbreviation: PSA: prostate-specific antigen.

## Discussion

While PSA testing has been used for decades for detecting early prostate cancer and monitoring therapy response, PSA levels are significantly affected by age, hypertrophy of the prostate, environmental factors, geography, diet and ethnicity. Thus, the age-specific PSA reference ranges proposed by Oesterling et al. [7] might not be applicable across all countries and ethnic groups. Studies from some Asian countries such as Korea, Japan and China suggest that, for all age groups, serum PSA levels in Asian people are lower than in other ethnic groups. However, Lin et al. [15] found that serum age-specific PSA levels among Taiwanese men are higher than those in other Asian ethnicities in all age groups, and approximate the levels in African-American men and Caucasian American men. The current study seeks to determine optimal age-specific PSA reference ranges for Taiwanese men based on a large cohort.

In this study, the median and 95% percentiles of serum PSA level in Taiwanese men are found to be directly related to age, and rise rapidly over the age of 50, consistent with results for different ethnic groups in different countries. Hypertrophy of the prostate can account for the elevation of serum PSA levels with age. However, over the age of 60, our subjects have higher age-specific PSA reference ranges than those observed by Oesterling et al. [7], which is contrary to the results for Japanese, Korean and Chinese subject Table 3.

**Table 3:** Comparison of serum PSA levels among African-Americans [21], Caucasian Americans [7], Asian-Americans [21], Taiwanese [15], Japanese [8], Koreans [11], and Chinese [12–14].

Age(year)	95th Percentile Value (ng/ml)									
	Current study	African- American	White- American	Asian- American	Taiwanese	Japanese	Korean	Chinese Shaanxi	Chinese Shanghai	Chinese Beijing
20–29	1.8	-	-	-	1.796	-	-	1.20	-	-
30–39	1.8	-	-	-	1.836	-	1.8	1.21	-	-
40–49	2.0	2.7	2.5	2.0	2.167	2.0	2.0	1.23	2.15	1.57
50–59	3.2	4.4	3.5	4.5	3.329	3.0	2.5	2.35	3.2	2.92
60–69	5.6	6.7	4.5	5.5	5.114	4.0	3.9	3.20	4.3	4.11
70–79	7.4	7.7	6.5	6.8	6.237	5.0	6.3	3.39	5.37	5.71

Abbreviation: PSA: prostate-specific antigen.

When compared with the age-specific PSA reference ranges from Oesterling et al., our results show that the difference in age-specific PSA reference ranges are relatively small for the 40-49-year-old age group (2.0 vs 2.5) and the 50-59-year-old age group (3.2 vs 3.5), but the direction of difference is inverted and obvious in the 60–69 (5.6 vs 4.5) and 70–79 (7.4 vs 6.5) year-old age groups Table 3. As a consequence, using the age-specific PSA reference ranges proposed by Oesterling et al. for screening of prostate cancer in Taiwan might result in errors by increasing sensitivity and decreasing specificity in patients over 60 years old. On the other hand, for those under 60 years old, errors in prostate cancer screening would still result from increased specificity and decreased sensitivity. From this study, the age-specific PSA reference ranges can be deduced to enhance the detection rate for prostate cancer in younger patients with PSA levels below the single cut-off of 4.0ng/ml and thus reduce the number of negative biopsies in older men with PSA levels > 4.0ng/ml [19]. Reissigl et al. [20] reported that using the age-specific PSA cut-off proposed by Oesterling et al. resulted in an 8% increase in the number of biopsies and an 8% increase in organ-confined cancer detection in men aged 45–59 years, while in older men aged 60–75 years, it resulted in about a 21% reduction in biopsies but would result in 4% of prostate cancers being missed.

Whether our results for age-specific PSA reference ranges are optimally appropriate for Taiwanese men is still open to question and the rate of accurate detection for prostate cancer remains to be determined. Further rigorous studies will be needed with tools including digital rectal examinations, transrectal ultrasound and prostate biopsy.

Our present results support the findings of a previous study for Taiwanese subjects by Lin et al [15], in which increasing age is found to produce higher PSA reference ranges than those of other Asian countries and near to the levels found in African-American men. Intriguingly, despite common ethnical origins with Chinese subjects, we note that serum age-specific PSA levels among men in Taiwan are higher than those in various regions of mainland China. The reason for this discrepancy remains unclear, though dietary and environmental factors may play a role [13].

The present study is subject to certain limitations. First, prostate biopsy, or transrectal ultrasound was not performed in participants with PSA levels > 4.0 ng/ml or abnormal digital examination. Thus the cohort could include prostate cancer cases, leading to increased PSA level reference ranges. Nevertheless, the large size of the cohort used in this study could reduce statistical bias. Second, most clients of the MJ Health Screening Center have a relatively high levels of disposable income, and thus lower economic class males may be underrepresented in the cohort.

## Conclusion

Serum PSA concentration increases with age, and the reference ranges of age-specific PSA levels in Taiwanese men are different from those in other ethnic groups and also in among ethnic Chinese in mainland China. The age-specific PSA reference ranges for Taiwanese men aged 60 and over are higher than those proposed by Oesterling et al. that are currently used in Taiwan and higher than those of other Asian countries. Age-specific PSA reference ranges should be population-specific. Our results may offer the important data to determine the age-specific PSA reference ranges in different age groups among Taiwanese men.

## References

[1] Oesterling JE. Prostate specific antigen: a critical assessment of the most useful tumor marker for adenocarcinoma of the prostate. J Urol. 1991;145(5):907–23. doi: 10.1016/s0022-5347(17)38491-4 1707989

[2] Catalona WJ, Smith DS, Ratliff TL, Dodds KM, Coplen DE, Yuan JJ, et al. Measurement of prostate-specific antigen in serum as a screening test for prostate cancer. N Engl J Med. 1991;324(17):1156–61. doi: 10.1056/NEJM199104253241702 1707140

[3] Oesterling JE. Age-specific reference ranges for serum PSA. N Engl J Med. 1996;335(5):345–6. doi: 10.1056/NEJM199608013350511 8663860

[4] Nadler RB. The case for prostate-specific antigen screening starting at age 40. Cancer. 2008;113(6):1278–81. doi: 10.1002/cncr.23721 18661528

[5] Moul JW, Sun L, Hotaling JM, Fitzsimons NJ, Polascik TJ, Robertson CN, et al. Age adjusted prostate specific antigen and prostate specific antigen velocity cut points in prostate cancer screening. J Urol. 2007;177(2):499–503; discussion -4. doi: 10.1016/j.juro.2006.09.063 17222618

[6] Thompson IM, Pauler DK, Goodman PJ, Tangen CM, Lucia MS, Parnes HL, et al. Prevalence of prostate cancer among men with a prostate-specific antigen level < or = 4.0 ng per milliliter. N Engl J Med. 2004;350(22):2239–46. 15163773 10.1056/NEJMoa031918

[7] Oesterling JE, Jacobsen SJ, Chute CG, Guess HA, Girman CJ, Panser LA, et al. Serum prostate-specific antigen in a community-based population of healthy men. Establishment of age-specific reference ranges. Jama. 1993;270(7):860–4. 7688054

[8] Oesterling JE, Kumamoto Y, Tsukamoto T, Girman CJ, Guess HA, Masumori N, et al. Serum prostate-specific antigen in a community-based population of healthy Japanese men: lower values than for similarly aged white men. Br J Urol. 1995;75(3):347–53. doi: 10.1111/j.1464-410x.1995.tb07347.x 7537604

[9] Masumori N, Tsukamoto T, Kumamoto Y, Miyake H, Rhodes T, Girman CJ, et al. Japanese men have smaller prostate volumes but comparable urinary flow rates relative to American men: results of community based studies in 2 countries. J Urol. 1996;155(4):1324–7. 8632564

[10] Morgan TO, Jacobsen SJ, McCarthy WF, Jacobson DJ, McLeod DG, Moul JW. Age-specific reference ranges for serum prostate-specific antigen in black men. N Engl J Med. 1996;335(5):304–10. doi: 10.1056/NEJM199608013350502 8663870

[11] Lee SE, Kwak C, Park MS, Lee CH, Kang W, Oh SJ. Ethnic differences in the age-related distribution of serum prostate-specific antigen values: a study in a healthy Korean male population. Urology. 2000;56(6):1007–10. doi: 10.1016/s0090-4295(00)00837-2 11113748

[12] He D, Wang M, Chen X, Gao Z, He H, Zhau HE, et al. Ethnic differences in distribution of serum prostate-specific antigen: a study in a healthy Chinese male population. Urology. 2004;63(4):722–6. doi: 10.1016/j.urology.2003.10.066 15072888

[13] Liu ZY, Sun YH, Xu CL, Gao X, Zhang LM, Ren SC. Age-specific PSA reference ranges in Chinese men without prostate cancer. Asian J Androl. 2009;11(1):100–3. doi: 10.1038/aja.2008.17 19050693 PMC3735217

[14] Liu X, Wang J, Zhang SX, Lin Q. Reference Ranges of Age-Related Prostate-Specific Antigen in Men without Cancer from Beijing Area. Iran J Public Health. 2013;42(11):1216–22. 26171333 PMC4499062

[15] Lin KJ, Pang ST, Chang YH, Wu CT, Chuang KL, Chuang HC, et al. Age-related reference levels of serum prostate-specific antigen among Taiwanese men without clinical evidence of prostate cancer. Chang Gung Med J. 2010;33(2):182–7. 20438671

[16] Wu X, Tsai SP, Tsao CK, Chiu ML, Tsai MK, Lu PJ, et al. Cohort Profile: The Taiwan MJ Cohort: half a million Chinese with repeated health surveillance data. Int J Epidemiol. 2017;46(6):1744–g. doi: 10.1093/ije/dyw282 28204597

[17] Lee SW. Regression analysis for continuous independent variables in medical research: statistical standard and guideline of Life Cycle Committee. Life Cycle. 2022;2:e3.

[18] Lee SW. Methods for testing statistical differences between groups in medical research: statistical standard and guideline of Life Cycle Committee. Life Cycle. 2022;2:e1.

[19] Polascik TJ, Oesterling JE, Partin AW. Prostate specific antigen: a decade of discovery—what we have learned and where we are going. J Urol. 1999;162(2):293–306. doi: 10.1016/s0022-5347(05)68543-6 10411025

[20] Reissigl A, Pointner J, Horninger W, Ennemoser O, Strasser H, Klocker H, et al. Comparison of different prostate-specific antigen cutpoints for early detection of prostate cancer: results of a large screening study. Urology. 1995;46(5):662–5. doi: 10.1016/S0090-4295(99)80297-0 7495117

[21] DeAntoni EP, Crawford ED, Oesterling JE, Ross CA, Berger ER, McLeod DG, et al. Age- and race-specific reference ranges for prostate-specific antigen from a large community-based study. Urology. 1996;48(2):234–9. doi: 10.1016/s0090-4295(96)00091-x 8753735

